# A Review of Atypical Cutaneous Histological Manifestations of Herpes Zoster

**DOI:** 10.3390/v16071035

**Published:** 2024-06-27

**Authors:** Maged Daruish, Gerardo Cazzato, Dorota Markiewicz, Saleem Taibjee, Francesco Fortarezza, Eduardo Calonje

**Affiliations:** 1 Department of Histopathology, Dorset County Hospital NHS Foundation Trust, London DT1 2JY, UK; saleem.taibjee@dchft.nhs.uk; 2 Section of Molecular Pathology, Department of Precision and Regenerative Medicine and Ionian Area (DiMePRe-J), University of Bari “Aldo Moro”, 70124 Bari, Italy; 3 Department of Dermatopathology, Guy’s and St Thomas’ NHS Foundation Trust, London DT1 2JY, UK; dorota.markiewicz@gstt.nhs.uk (D.M.); eduardo.calonje@gstt.nhs.uk (E.C.); 4 Surgical Pathology and Cytopathology Unit, University Hospital of Padova, 35100 Padova, Italy; francesco.fortarezza@aopd.veneto.it

**Keywords:** varicella-zoster virus, herpes zoster, atypical presentation

## Abstract

The clinical and histopathological features of herpes zoster (HZ) are usually straightforward. Atypical histological presentations, in the absence of the classical viral cytopathic changes, are well documented and can make the diagnosis of HZ extremely difficult. Herein, we review the existing literature on atypical cutaneous histological manifestations of the disease, with emphasis on the subtle clues, use of immunohistochemistry, and potential pitfalls.

## 1. Introduction

Varicella-zoster virus (VZV) is a double-stranded DNA virus that belongs to the alpha-herpesvirus family [1,2]. Primary infection with VZV is responsible for varicella (chickenpox), while reactivation of the virus latent in the dorsal root ganglia results in herpes zoster (HZ) or shingles [2].

**Figure 1:**
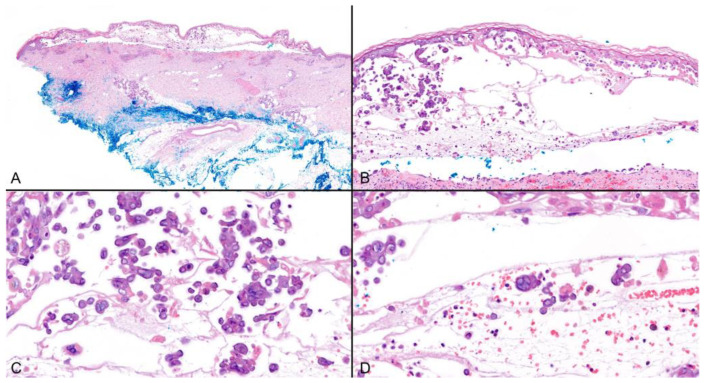
**Typical histology of cutaneous VZV infection:** (**A**,**B**) Suprabasal separation consequent to ballooning, reticular degeneration, and keratinocytes necrosis; hematoxylin and eosin (H&E) ×10 and ×100. (**C**) Steel grey nuclei with peripheral condensation of chromatin can be seen; (H&E) ×200. (**D**) Multinucleated cells are common; (H&E) ×200.

HZ (shingles) clinically presents as a painful vesicular rash in a dermatomal distribution, more commonly in the older population [3]. Although typical cases are rarely biopsied, the histological features tend to be similar to those of herpes simplex (HSV) infections, including secondary acantholysis of keratinocytes as a result of ballooning, and resulting in intraepidermal separation, reticular degeneration, and epithelial necrosis (Figure 1A,B). Nuclear viral-induced changes are characteristic, including chromatin margination, steel grey nuclei (ground glass appearance), and multinucleation. Intranuclear eosinophilic inclusions may be seen (Figure 1C,D) [4].

**Figure 2:**
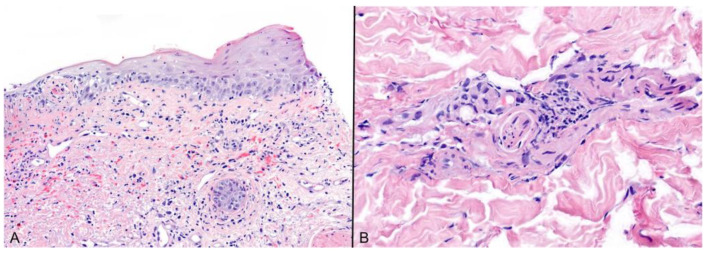
**Subtle changes in HZ:** (**A**,**B**) Interface vacuolar damage, erythrocytes extravasation, and perineural inflammation may be the only pathological features identified; (H&E) ×200.

**Figure 3:**
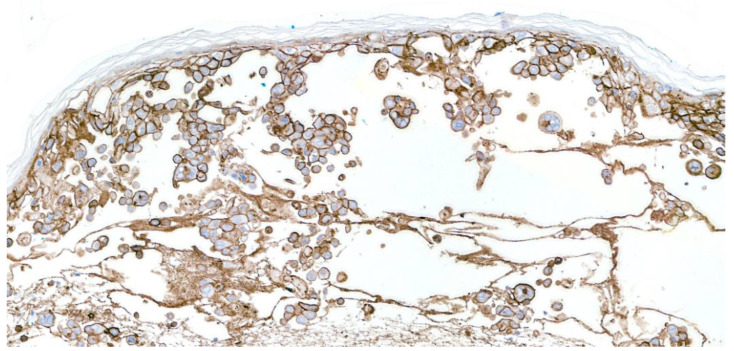
IHC for VZV shows diffuse and strong membranous staining in active lesions ×200.

HZ, however, can manifest without the typical clinical and histological appearances (i.e., herpes incognito), particularly in immunosuppressed patients, posing diagnostic challenges [5,6]. In these instances, scrutiny for focal pathognomonic changes in serial sections may help reach the diagnosis [5]. Furthermore, the epidermal cytopathic changes may be completely lacking with only subtle clues of VZV infection seen (Figure 2), including interface changes, red blood cells extravasation, neuritis, and sebaceitis [5,6,7]. In the authors’ experience, a larger incisional biopsy can be beneficial when looking for the latter features. Ancillary studies, such as immunohistochemistry (IHC), PCR, and viral cultures can be of extreme value in confirming VZV infection in challenging cases [8,9]. IHC for herpes simplex should not be used if VZV is suspected, as there is no cross-reaction. VZV IHC has high sensitivity and specificity and shows membranous staining in the productive phase of the infection (Figure 3), while in more prolonged infections it has a nuclear and perinuclear positivity pattern [10,11].

In this review, we discuss the clinical and histopathological features of the cutaneous manifestations of HZ, focusing on the atypical/rare presentations.

## 2. Materials and Methods

We conducted an extensive literature review on PubMed using the terms ‘atypical herpes zoster histology’, ‘herpes folliculitis’, ‘herpes syringitis’, ‘herpes vasculitis’, ‘verrucous herpes’, and ‘pseudolymphoma’. Papers describing histological features of skin manifestations involving sites of previous herpes zoster infections were excluded. Other excluded papers were those that were not available in the English language, and papers describing manifestations related to herpes simplex viruses, or in which the type of infecting virus was not confirmed by ancillary modalities such as PCR or IHC.

## 3. Atypical VZV Presentations

### 3.1. Varicella-Zoster Folliculitis

Involvement of the hair follicle epithelium in biopsies of classic herpes zoster lesions is not uncommon [12,13]. However, exclusive folliculitis in absence of epidermal changes appears to be rare and possibly under-recognized [6,7,14,15]. Herpetic folliculitis is more commonly reported as a manifestation of VZV rather than HSV [6,16]. It is believed that the reactivated VZV travels through the sensory myelinated nerves terminating at pilosebaceous units, in contrast to HSV that travels via the non-myelinated twigs innervating the skin [6,7,12].

VZV folliculitis is diagnostically challenging both clinically and histologically. The usual vesicles are typically absent, and the clinical picture is broad, including follicular-based erythematous papules, erythematous patches, plaques, pustules, nodules, and purpura [6,7,14,15,17,18]. In addition, the lesions can be disseminated or lack a clear-cut segmental distribution [6,18]. Patients presenting with VZV folliculitis tend to be immunocompromised with known HIV, hematopoietic or solid malignancies, or other causes of immunosuppression [15,16,18,19]. Reports in immunocompetent patients also exist [7,14]. As mentioned above, involvement of the pilosebaceous units only with sparing of the epidermis may be the only histological sign of the disease.

**Figure 4:**
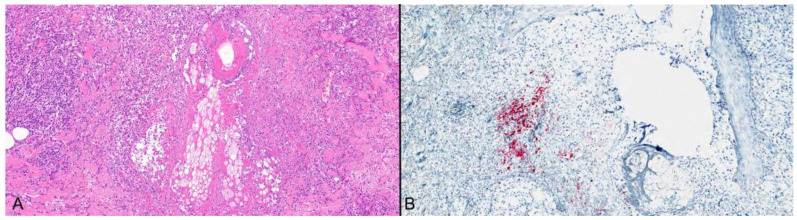
(**A**) Necrotizing VZV folliculitis; (H&E) ×200. (**B**) Nuclear staining for VZV IHC in follicular epithelial remnants; (H&E) ×200.

Cytopathic changes can be present, particularly in the follicular isthmus and sebaceous glands in subtle cases [6,16]. These changes, however, can also be focal or completely lacking. In other cases, individual, or en masse follicular keratinocytic necrosis may be prominent (Figure 4) [6,7,14,15]. Extravasation of erythrocytes and dense perifollicular lymphoid inflammation have been described as commonly associated features [6,14,16]. The lymphocytic inflammatory cell infiltrate may be misinterpreted as cutaneous lupus erythematosus [7], rosacea [5], or masquerade as a lymphoma (also see below), particularly if there is cytological atypia as a result of the presence of activated T-cells [13].

### 3.2. Pseudolymphoma Associated with Herpes Zoster

Leinweber et al. [13] described T-lymphocyte-predominant infiltrates mimicking lymphoma in PCR-confirmed herpes simplex and herpes zoster (in 31 patients of the latter). Lymphocytic atypia was observed in most of the specimens examined with some displaying severe cytological atypia. Adding to the diagnostic difficulty, CD30+ T-lymphocytes may be present in variable numbers and can form clusters, a finding that can also be observed in various infections and inflammatory conditions. In this scenario, differentiation from primary cutaneous CD30+ lymphoproliferative disorders may be challenging [5,13,20,21,22].

**Figure 5:**
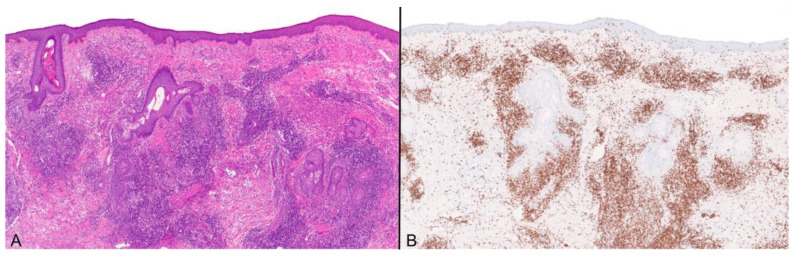
(**A**) Dense perifollicular lymphoid cell infiltrate in the absence of viral cytopathic changes in the epidermis and follicular epithelium; (H&E) ×100. (**B**) The lymphocytes are T-cells as confirmed by CD3 diffuse staining ×100. Necrosis and cytopathic changes may be found after numerous serial sections are examined.

As previously discussed, VZV folliculitis can be accompanied by a dense perifollicular lymphoid inflammatory cell infiltrate (Figure 5) [6,14,16]. Three of the herpes zoster pseudolymphomatous cases in Leinweber’s series lacked epidermal involvement and the lymphoid infiltrates were closely associated with the pilosebaceous units [13]. In one case mimicking CD30+ anaplastic large cell lymphoma, necrotic follicular keratinocytes and multinucleated giant cells were only identified on closer scrutiny, after the clinical picture evolved to become more typical [22].

### 3.3. Herpetic Syringitis

Typical viral cytopathic changes involving the eccrine epithelium (herpetic syringitis) have rarely been described in herpes zoster, particularly in the setting of HIV infection [23,24]. Additional reported cases include patients with lymphoma and one patient with inflammatory bowel disease on biologics [25,26,27]. Alonso-Perez et al. speculated that herpetic syringitis may be underdiagnosed [26]. Herpetic syrngitis may represent the only clue to the diagnosis, with the overlying epidermis either unaffected or extensively necrosed [23,24,26].

### 3.4. Varicella-Zoster Vasculitis

There has been increasing evidence in the literature linking VZV reactivation to cerebral vasculitis and stroke, the classic example being herpes zoster ophthalmicus followed by ipsilateral cerebral artery infarction and contralateral hemiparesis [28,29,30,31]. Herpes zoster presenting as cutaneous small vessels vasculitis has been reported rarely, including in patients with sarcoidosis, cutaneous T-cell lymphoma, and on immunosuppressive medications [32,33,34,35,36,37,38,39]. Direct VZV infection of the endothelium has been demonstrated by immunohistochemistry and also by electron microscopy [32,33,34]. Potential routes hypothesized include direct spread from adjacent dermal nerves, the overlying epidermis (if involved), or through hematogenous spread with low-grade viremia [32,35,36].

**Figure 6:**
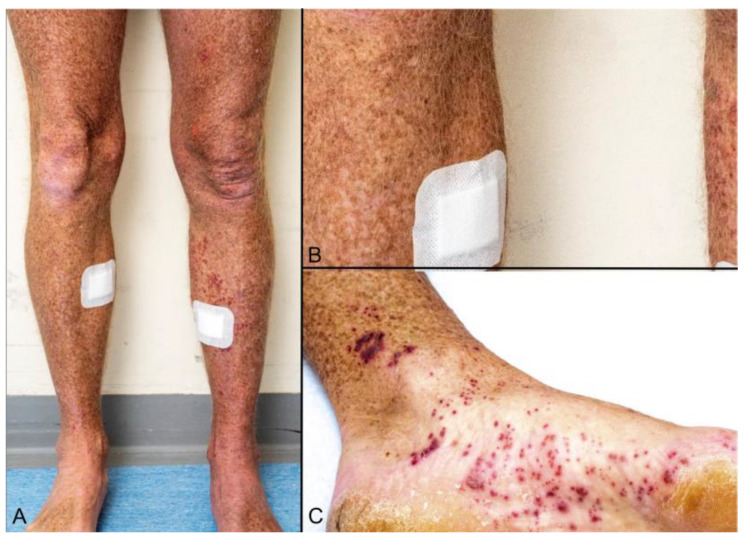
A rare clinical presentation of bilateral vasculitic HZ in an immunosuppressed patient. He also had radiculopathy with leg weakness (mimicking Guillain–Barré neuropathy). This all resolved with acyclovir.

Clinical manifestations of VZV-induced vasculitis described include palpable purpura, erythematous to violaceous painless nodules, and necrotic ulcers. Typical zoster vesicles can be absent. However, the segmental distribution of the vasculitic lesions may be a clue to the diagnosis [32,33,34,35,36,37,38,39]. Presentations with a bilateral distribution (Figure 6) or in immunocompetent patients have been exceptionally reported [33,37].

**Figure 7:**
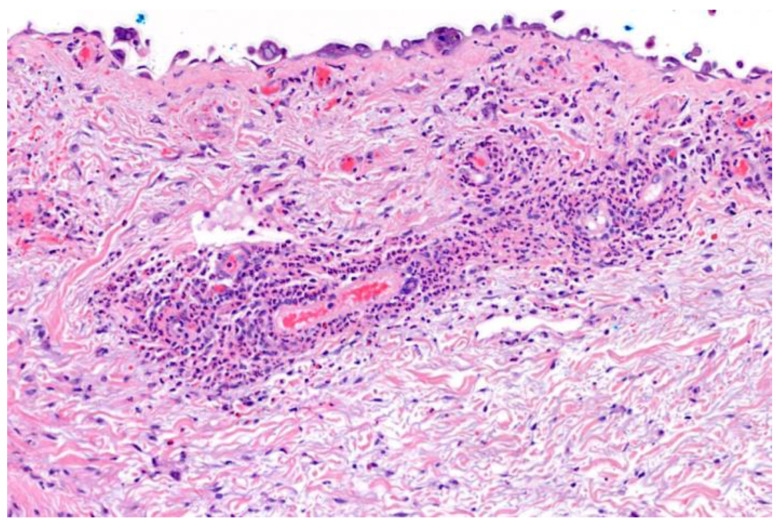
Leucocytoclastic vasculitis underlying a blister with VZV cytopathic changes; (H&E) ×200.

Histologically, these lesions tend to show evidence of leucocytoclastic vasculitis (Figure 7) [32,33,36,37,38,39], although lymphocytic vasculitis has also been described [34,35]. VZV-induced vasculitis in absence of epidermal viral cytopathic changes is extremely rare [33,34].

### 3.5. Verrucous Varicella-Zoster Infection

Chronic VZV infection has been reported to mimic squamoproliferative lesions clinically [10]. Verrucous lesions are more commonly seen in VZV in comparison to HSV infections, with no specific site distribution [10,40]. The lesions tend to present as a single, less commonly multiple, vegetating, hyperkeratotic plaque with rare ulceration [10,40]. The exact pathogenesis behind the verrucous morphology is unclear. One possibility is altered viral tyrosine kinase expression or L protein in a background of immune dysregulation [40]. This is mostly seen in persistent VZV infection (at least one-month duration) in HIV patients and in iatrogenic immunosuppression [10,40,41,42,43]. Presentation in immunocompetent patients is rare [44].

Histological examination usually shows orthokeratotic hyerperkatosis with columns of parakeratosis. Pseudoepitheliomatous hyperplasia may be seen. Cytolytic changes are variable and may be absent or identified only focally, possibly at the edge of the lesion [10,40]. In one case, the multinucleated cells were present in the follicular isthmus [18]. A VZV monoclonal antibody is useful in this setting and displays a nuclear and perinuclear pattern of staining [10,40].

### 3.6. Isotopic Response, Koebner’s Phenomenon, and Renbök’s Phenomenon

An interesting paper explored the unusual presentation of secondary skin diseases in three patients with pre-existing HZ and analyzed the immunological and pathological mechanisms underlying these manifestations, discussing various phenomena such as the isotopic response, Koebner’s phenomenon, and Renbök’s phenomenon. With regard to the isotopic response, this is the appearance of a new skin disease at the site of a previously healed skin and, in this paper, the third patient presented developed lichen planus following an acute episode of HZ. Potential mechanisms include long-term immune dysregulation, nerve damage, neuropeptide secretion, altered blood flow, and/or scarring in the affected area. Koebner’s phenomenon is the development of skin lesions in response to trauma in an otherwise unaffected area. The first patient presented showed Wegener’s granulomatosis (WG) lesions triggered by HZ acting as a localized trauma. Finally, Renbök’s phenomenon represents the event in which normal hair growth is observed in psoriatic plaques of patients with alopecia areata and, in this regard, the second patient developed a cutaneous graft-versus-host disease (GVHD) months after the HZ, with immune changes in the dermatome preventing GVHD [45].

## 4. Conclusions

Unusual histopathological findings can render the diagnosis of HZ difficult, and the histopathologist should have a high index of suspicion in biopsies of immunosuppressed patients. The typical epidermal changes can be entirely absent or be non-specific with interface damage only. One should consider serial sections or advise further biopsies when necessary. Potential patterns encountered include inflammation or necrosis of the hair follicles, eccrine epithelium, vasculitis, pseudolymphoma, and pseudoepitheliomatous hyperplasia. IHC for VZV can be invaluable to confirm the diagnosis.
